# Ultrasound-based assessment of the expression of inflammatory markers in the rectus femoris muscle of rats

**DOI:** 10.3389/ebm.2024.10064

**Published:** 2024-02-29

**Authors:** Bahareh Ahmadi, Felipe C. K. Duarte, John Srbely, Pawel M. Bartlewski

**Affiliations:** ^1^ Department of Biomedical Sciences, Ontario Veterinary College, University of Guelph, Guelph, ON, Canada; ^2^ School of Health, Medical and Applied Sciences, Central Queensland University, Brisbane, QLD, Australia; ^3^ Department of Human Health and Nutritional Sciences, College of Biological Sciences, University of Guelph, Guelph, ON, Canada

**Keywords:** rat, neurogenic inflammation, rectus femoris, inflammatory regulators, first order echotextural variables

## Abstract

Ultrasonographic characteristics of skeletal muscles are related to their health status and functional capacity, but they still provide limited information on muscle composition during the inflammatory process. It has been demonstrated that an alteration in muscle composition or structure can have disparate effects on different ranges of ultrasonogram pixel intensities. Therefore, monitoring specific clusters or bands of pixel intensity values could help detect echotextural changes in skeletal muscles associated with neurogenic inflammation. Here we compare two methods of ultrasonographic image analysis, namely, the echointensity (EI) segmentation approach (EI banding method) and detection of selective pixel intensity ranges correlated with the expression of inflammatory regulators using an in-house developed computer algorithm (r-Algo). This study utilized an experimental model of neurogenic inflammation in segmentally linked myotomes (i.e., rectus femoris (RF) muscle) of rats subjected to lumbar facet injury. Our results show that there were no significant differences in RF echotextural variables for different EI bands (with 50- or 25-pixel intervals) between surgery and sham-operated rats, and no significant correlations among individual EI band pixel characteristics and protein expression of inflammatory regulators studied. However, mean numerical pixel values for the pixel intensity ranges identified with the proprietary r-Algo computer program correlated with protein expression of ERK1/2 and substance P (both 86–101-pixel ranges) and CaMKII (86–103-pixel range) in RF, and were greater (*p* < 0.05) in surgery rats compared with their sham-operated counterparts. Our findings indicate that computer-aided identification of specific pixel intensity ranges was critical for ultrasonographic detection of changes in the expression of inflammatory mediators in neurosegmentally-linked skeletal muscles of rats after facet injury.

## Impact statement

Early detection of biochemical changes in skeletal muscles could greatly improve the diagnosis and treatment of various myopathies. There is a great deal of evidence to suggest that ultrasonographic characteristics of skeletal muscles can be used to determine their chemical composition. The main objective of the present experiment was to use computer-assisted image analysis of muscle ultrasonograms to estimate the content of neuroinflammatory regulators. We have designed and written a computer algorithm (r-Algo) to identify the specific ranges of echointensity values with the strongest correlation to the biochemical constituents of muscles in rats following the experimental facet injury. Mean echointensity values for r-Algo−detected pixel ranges (associated with the expression of affected inflammatory regulators) differed significantly between the surgery and sham-operated animals. These results indicate that r-Algo provides a novel and effective method of quantitative image analysis, with multiple potential applications in biomedical research, medicine, and industry.

## Introduction

Muscle inflammation plays a significant role in the etiology of many diseases, including primary inflammatory myopathy and several muscular dystrophies [1]. Various approaches are available for diagnosing and monitoring muscle inflammation, including magnetic resonance imaging (MRI), computed tomography (CT), and skeletal muscle biopsies; however, these techniques are invasive (muscle biopsies), expose the individual to ionizing radiation (CT), or have limited accessibility (MRI) [2, 3]. Still, developing a non-invasive or minimally invasive method for detecting and monitoring microstructural and biochemical alterations during myositis (idiopathic or neurologic) has lagged behind [1, 4].

Ultrasound imaging (grey-scale B-mode) is a safe diagnostic method that permits visualization of internal organs and tissues with relatively high spatial resolution [5]. Its advantages can mainly be attributed to “real-time” capabilities and the fact that it enables the clinicians to sample patients with much greater frequency than with other imaging techniques, thereby providing greater sensitivity/specificity to the diagnosis and treatment responses of patients [4, 5]. This technique is increasingly used for soft tissue evaluations in a wide range of medical fields [4]. However, the interpretation of ultrasonographic findings is heavily dependent on the diagnosticians’ expertise and image quality. Furthermore, B-mode image echointensity is variable and likely dependable on the machine/system settings and operator, making comparisons across diagnostic centers difficult and inconsistent [4]. In 1989, Heckmatt et al. [6] developed quantitative sonography of skeletal muscles, which is less dependent on operator’s experience as well as more objective and accurate compared with visual inspection of ultrasonograms. Using this approach, muscle echotextural features are computed within a specific region of interest (ROI) [6]. Since then, numerical echo intensity (EI) values have become the most commonly used ultrasound-based measure of skeletal muscle status [7–10]. Several ensuing studies confirmed that muscle EI was a useful and practical surrogate measure of skeletal muscle health and functional capacity [7–10], particularly in diagnosing and evaluating the progression of numerous neuromuscular disorders associated with the inflammatory process [11]. However, EI still provides limited information on muscle chemical constituents [12]. The biochemical composition of skeletal muscles is known to change during the development of pathological conditions [1, 4]. Therefore, it is important to describe the accompanying echotextural changes in order for transcutaneous ultrasonography to become a useful technique in detecting and frequently monitoring muscle inflammation. Recently, Pinto and Pinto [13] observed that EI capturing a complete range of pixel intensities or numerical pixel values of the organ of interest (ranging from 0 (absolute black) to 255 (absolute white)) might convey inadequate information on their chemical composition and/or the extent of histomorphological changes. Therefore, assessing EI parameters and pixel distribution within different EI clusters of the grey-scale histogram may provide more accurate information on muscle internal characteristics [13]. New ultrasound image processing and analysis methods are urgently needed to obtain critical information on tissue chemical composition.

Given these lines of evidence, we developed a computer algorithm (provisionally called r-Algo). First, the program screens the numerical pixel values in the images/specified ROIs. Next, it identifies the conglomerates or groups of pixel values for which the linear correlations between echogenic and other quantitative characteristics of the tissue (including those determined *ex situ* with various laboratory techniques) are strongest (based on the value of correlation coefficient-r). The r-Algo calculates mean numerical pixel values for each possible combination of pixels in the image bitmap (with luminance values ranging from 0 to 255), and computes correlation coefficients between pixel intensity for all pixel ranges/clusters (input variables) and any equinumerous data set (output variables), using the Pearson Product moment analysis. Lastly, the software reports a continuous pixel range or a scattered group of pixels for which the strongest linear correlation between the two sets of quantitative data (echotextural and other) has been identified.

The aim of this work was to develop methodological and statistical criteria whereby the linear association between the combination of nominal pixel values and protein expression of inflammatory mediators in the affected skeletal muscles of rats could be established. In addition, we applied the r-Algo program to: i. assess the suitability of using the predictive pixel intensity ranges for detecting differences in the expression levels of inflammatory mediators; and ii. compare this approach with the EI banding method using two “arbitrary” pixel ranges most commonly employed in the past studies (with the 50- and 25-pixel intervals). The existence of such quantitative relationships would greatly enhance the sensitivity and specificity of this clinically-feasible diagnostic approach (ultrasound imaging combined with computer-aided image analysis) for the monitoring and management of inflammatory muscle disease.

## Materials and methods

### Experimental procedures

**FIGURE 1:**
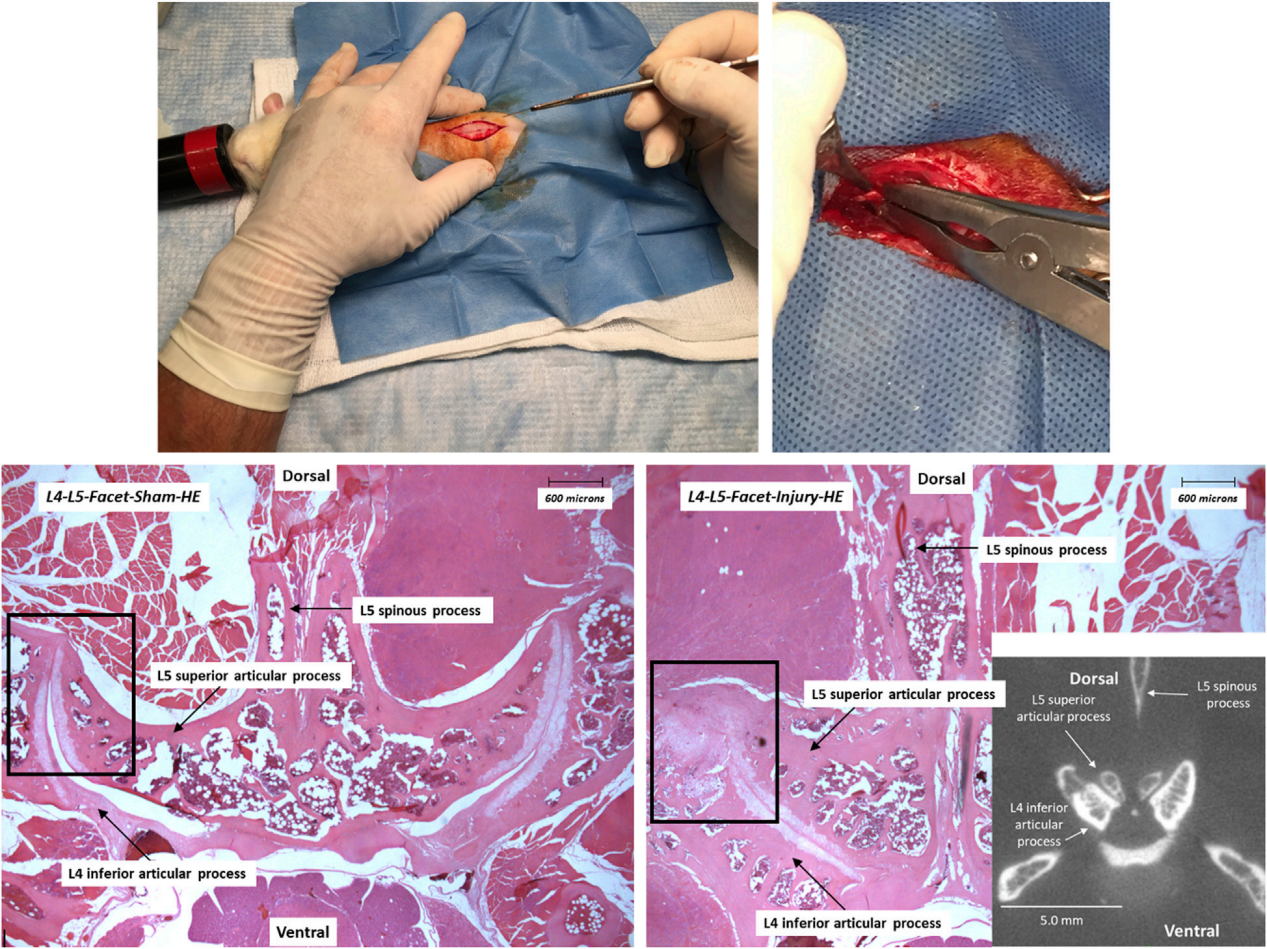
Experimental procedures (upper panels; scalpel incision and left L4-L5/L5-L6 joint compression using modified Kelly forceps) and histopathological (hematoxylin-eosin (HE) staining)/radiological (micro-CT) evaluations of the lumbar facet articular joint injury (L4-L5; lower panels) 21 days after surgical procedures in sham-operated (left) and surgery (right) rats. Targeted injury sites are delineated with rectangles. Photographic reproductions of histograms and a micro-CT scan insert show the intact L4-L5 facet joint in a sham-operated rat and the injured facet in a surgery group animal.

The Animal Care Committee of the University of Guelph (Guelph, ON, Canada) had approved all experimental procedures involving live animals. The present retrospective study used the original data and ultrasound images obtained and analyzed by Duarte et al. [14] and Ahmadi et al. [15], but with the new discriminating image analysis of muscle ultrasonograms. Twelve sexually mature male Wistar Kyoto rats, aged 13 ± 5 months, were housed (2–3 animals per cage) in a room with a 12-hour alternating light-dark cycle and a stable temperature (23.0 ± 1.0°C). All animals received pellet diet *ad libitum*. Animals were divided into two groups: surgery (animals that subsequently underwent facet compression at lumbar segments L4-L6 on the left side; Figure 1), and sham-operated (animals with left side facets exposed but without facet compression) [16, 17]. A non-steroidal anti-inflammatory drug carprofen (5 mg/kg body mass) was administered subcutaneously 30 min before surgical procedures. Both surgery and sham animals were anesthetized using 4% isoflurane and had a local anesthesia applied over the incision site (2–5 mg/kg of 50/50 lidocaine/bupivacaine). Once animals were anesthetized, a posterior midline incision of skin and subcutaneous tissue was made along the L2 to L6 spinous processes. A lumbar muscle at the L3-L4-L5 spinous process (multifidus) was resected to expose the lumbar facet capsule. In the experimental facet surgery group, the left L4-L5 and L5-L6 facet joints were then compressed with modified Kelly forceps. All muscles were sutured (braided 4-0 coated vicryl) and the skin was closed using stainless-steel staples. After regaining consciousness, rats were returned to their cages and maintained in the same conditions as prior to surgical manipulations. The effectiveness of surgical procedures was verified 21 days later with histopathological examinations and micro-CT scans (Figure 1).

### Ultrasound scanning and image acquisition

**FIGURE 2:**
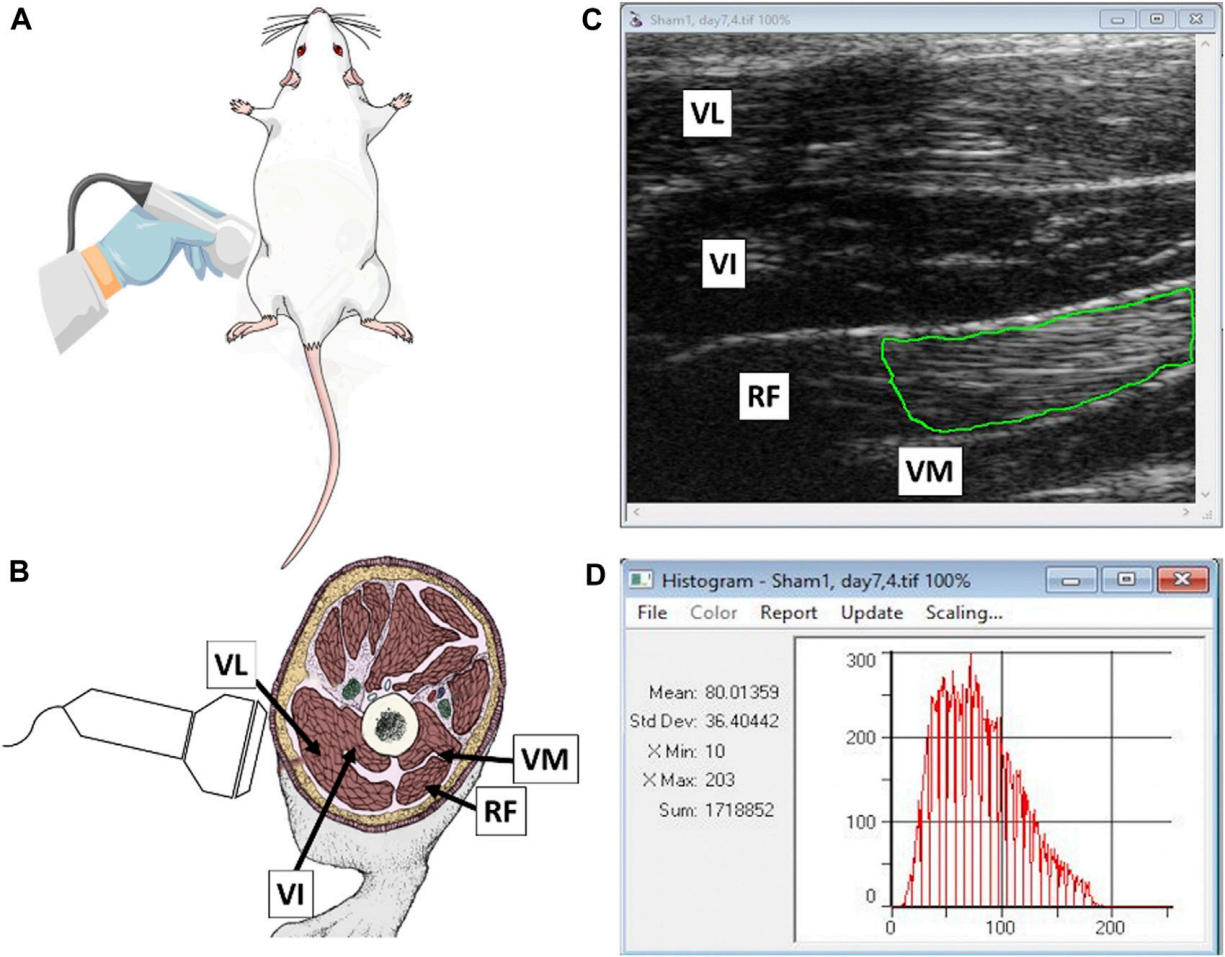
**(A)** Schematic illustration of the scanning technique employed to visualize hindlimb muscles of experimental rats; **(B)** topographic location of vastus lateralis (VL), vastus intermedius (VI), rectus femoris (RF) and vastus medialis (VM) muscles; **(C)** a normalized ultrasonogram containing individual muscles with a green line illustrating a possible positioning of a polygonal region of interest (ROI) used for determining echotextural characteristics of RF parenchyma; and **(D)** a summary of digital image analysis performed with ImageProPlus^®^ computer software (Mean, mean numerical pixel values; Std Dev, pixel heterogeneity; X Min and X Max, minimal and maximal pixel intensity values within ROI; and Sum, a total number of pixels encapsulated by an ROI; a red graph is a pixel distribution histogram with pixel intensity values along the *x*-axis and the numbers of detected pixels of specific intensities on a *y*-axis).

Transcutaneous ultrasonography of various muscle groups in the rats of the present sudy has been detailed previously [14, 15]. All ultrasound images of the rectus femoris muscle 21 days after surgical interventions were recorded in B-mode using the Vevo 2100 Visuals Sonic Ultrasound Diagnostic Medical Imaging System (Visual Sonic Inc., Toronto, ON, Canada; Figure 2). The equipment settings for transducer frequency (24 MHz), overall and near/far gain, contrast, and focal points were kept constant throughout the entire study. To eliminate a scale bar and all typed characters in the image text area, digital images were cropped, converted to grayscale images with a bit depth of eight ranges and pixel intensity from 0 (absolute black) to 255 (absolute white), and then normalized using a r-Algo and the following formula: G_i_ = T (f_i_−f_min_)/(f_max_−f_min_), where f_i_ was the original intensity in the range (f_min_, f_max_) and G_i_ was the corresponding scaled intensity to lie within (0,T).

### Western blotting of inflammatory regulators

All animals were euthanized with carbon dioxide on Day 21 of the experiment (Day 0 = surgery) and the samples of the rectus femoris were collected for Western blot analyses of inflammatory regulators, as described previously by Ahmadi et al. [15]. Muscle samples (each 20–30 mg) were frozen and then homogenized with cell lysis buffer (NP40 CLB-FNN0021; Thermo Scientific Fisher, Canada) containing protease inhibitor and serine protease inhibitor phenylmethylsulphonyl fluoride (PMSF). A copper-based assay using bicinchoninic acid (BCA) protein detection kit (Pierce BCA Protein Assay; Thermo Scientific, Canada) was used to determine protein content in the homogenized samples [18]. Equal amounts of muscle sample proteins (15 μg) were separated by sodium dodecyl sulfate-polyacrylamide gel (15%) electrophoresis and transferred for 1 h at 0.2 A onto a polyvinylidene difluoride membrane. Membranes were blocked in 5% non-fat skim milk tris-buffered saline (TBS-T) containing 0.1% tween-20 for 1 h at room temperature followed by an overnight incubation at 4°C with primary antibodies diluted in 5% bovine serum albumin. The primary antibodies used were as follows: anti-substance P antibody at 1:500 (orb215527), anti-calcitonin-gene related peptide (CGRP) 1:250 (orb182870) and anti-protease-activated receptor-2 (PAR2) antibody at 1:500 (orb385619); Biorbyt Ltd., San Franciso, CA, United States; anti-proline-directed kinases (ERK1/2) antibody at 1:1,000 (#9102); and anti-Ca^2^⁺/calmodulin-dependent protein kinase II (CaMKII) antibody at 1:1,000 (#4436); Cell Signaling Technology, Denver, MA, United States. After three washes in TBS-T, the membranes were incubated at room temperature for 1 h with the secondary antibody (anti-rabbit—1:2000, A0545; Sigma-Aldrich, Oakville, ON, Canada). Protein bands were detected using enhanced chemiluminescence (PerkinElmer; Waltham, MA, United States) and relative protein content was quantified by densitometry using a chemiluminescence detection system (Alpha Innotech Fluorchem HD2, Fisher Scientific, Hampton, NH, United States); all results were normalized against the loading control (alpha-tubulin protein, ab7291; Abcam, Cambridge, MA, United States).

### Image analysis based on arbitrarily chosen clusters of echointensity values (EI bands)

The regions of interest in the images containing the largest cross-sectional area of the rectus femoris were delineated using a polygonal tool and subjected to the bitmap analysis using ImageProPlus^®^ analytical software (Media Cybernetics Inc., Rockville, MD, United States). Bitmap analysis organizes and displays the values of individual pixels in a bitmapped image/rectangular ROI. Using the “Save” command on the “Histogram and Bitmap Analysis” file tab, the results of the analysis were stored in a file for processing with other applications at a later date. All tabulated pixels from the grayscale (B-mode) matrix were exported to a customized Excel spreadsheet to calculate the mean pixel intensity (numerical pixel values-NPV) and heterogeneity (standard deviation (SD) of mean numerical pixel values) in the ranges of: i. 0–50, 51–100, 101–150, 151–200, and 201–255 or 0–25, 26–50, 51–75, 76–100, 101–1,025, 126–150, 151–175, 176–200, 201–225, 226–250, and 251–255.

### Image analysis using in-house Python algorithm r-Algo

**FIGURE 3:**
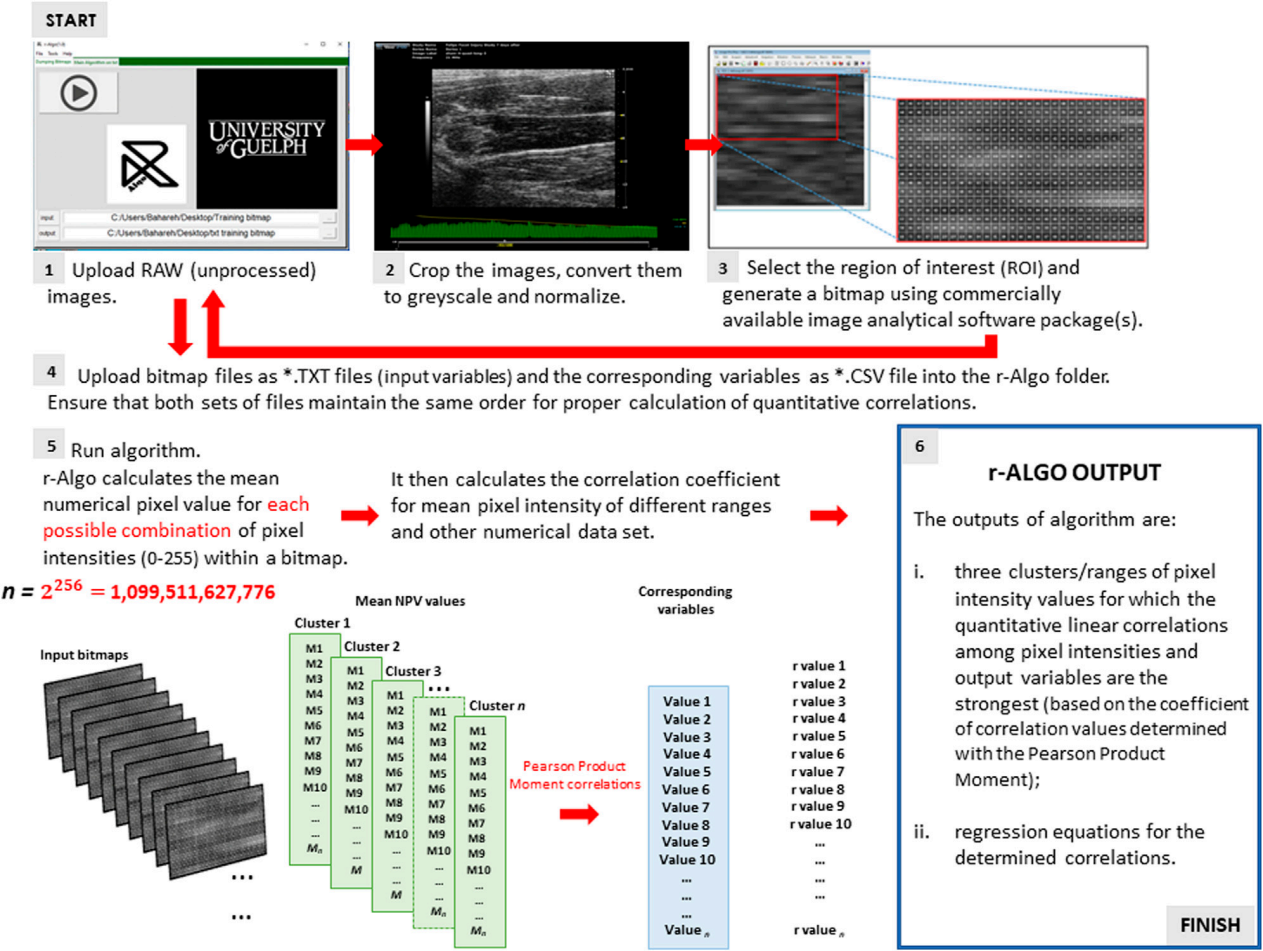
A simplified r-Algo (computer algorithm) operating flow chart.

The mean value for pixel intensities for each possible combination of individual pixel values (0–255) within each bitmap ( $2^{256}=$ 1,099,511,627,776) were calculated by r-Algo. The program also computed coefficients of correlation between averaged values for all pixel value combinations/clusters (input variables) and the expression of inflammatory mediators (output variables: Figure 3). Lastly, the r-Algo identified a pixel range with the strongest linear correlation (highest correlation coefficient) for each output variable.

### Statistical comparisons and correlational analyses

All statistical tests were completed using the SigmaPlot^®^ program (Systat Software Inc., San Jose, CA, United States). The percentages of pixels in different numerical pixel value ranges (EI bands 50 and 25) as well as the differences in mean pixel intensity and heterogeneity values between the two groups of animals (surgery and sham-operated) were analyzed for each EI range by Student *t*-test. Similarly, pixel distribution, as well as mean pixel intensity and heterogeneity values for specific pixel ranges identified with the r-Algo, were compared between surgery and sham-operated rats by Student *t*-test. Correlational analyses between first order echotextural variables or pixel frequencies/distribution and the expression of inflammatory regulators utilized the Pearson Product Moment test. *p*-value < 0.05 was considered statistically significant and all results are expressed as mean ± standard error of the mean (SEM).

## Results

**FIGURE 4:**
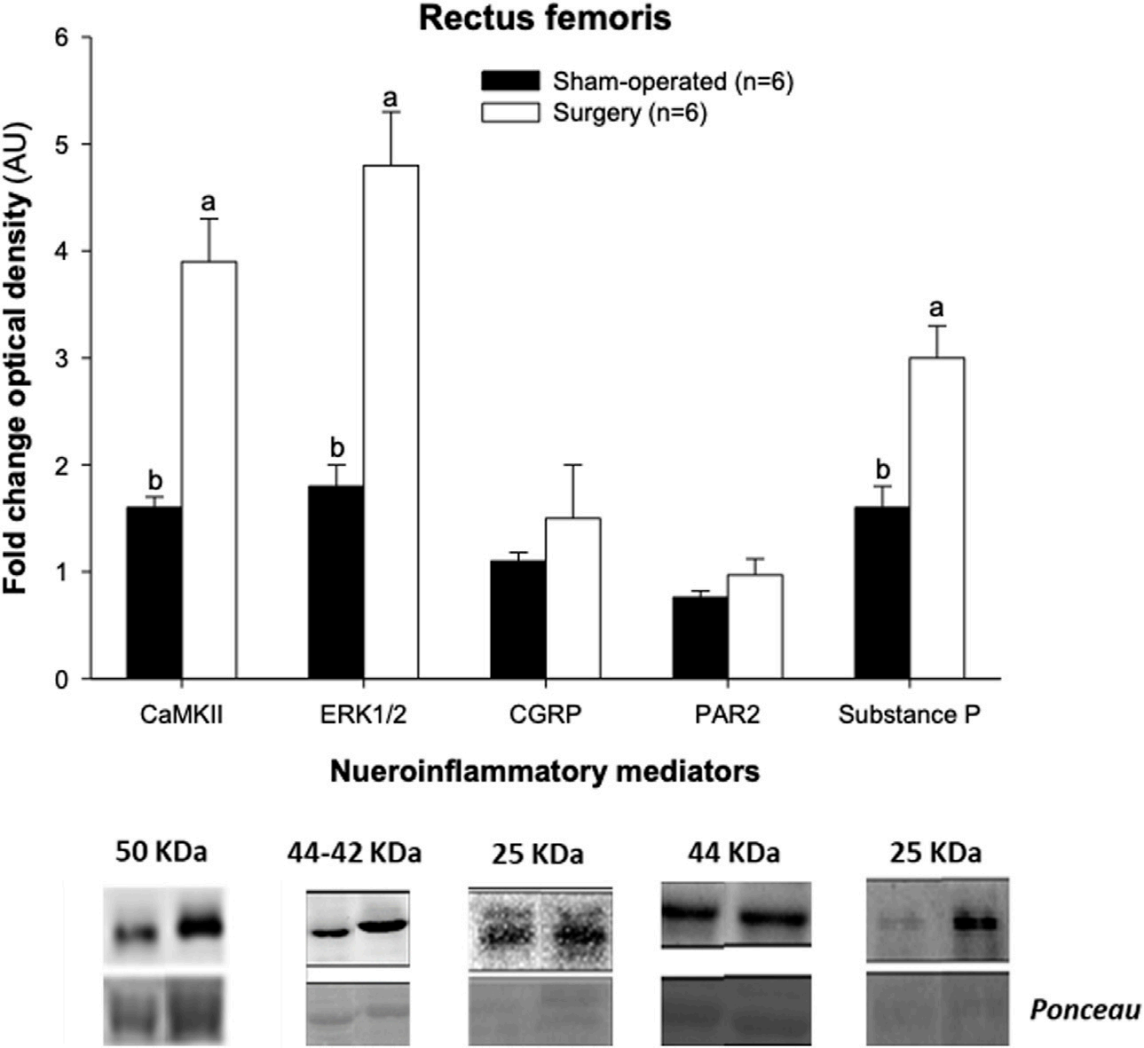
Relative protein expression of inflammatory mediators determined in left rectus femoris homogenates obtained post-mortem 21 days after experimental procedures from sham-operated and experimental surgery (lumbar L4-L6 facet injury) Kyoto Wistar rats. Ponceau is the generic name for a family of azo dyes used for gel staining. CaMKII, Ca^2^⁺/calmodulin-dependent protein kinase II; ERK1/2, proline-directed kinases; CGRP, calcitonin-gene related peptide; and PAR2, protease-activated receptor-2. ab, *p* < 0.05.

Western blot studies revealed an occurrence of a 2.4-fold, 2.7-fold and 1.9-fold rise in CaMKII, ERK1/2 and substance P concentrations, respectively, in the rectus femoris of surgery rats compared with sham-operated animals (Figure 4). No effect of facet injury on the protein expression of CGRP or PAR2 in the rectus femoris was noted on Day 21 after initial experimental interventions (*p* > 0.05). The mean pixel intensity for the rectus femoris muscle did not vary significantly between surgery and sham-operated groups of experimental rats (67.5 ± 8.1 compared with 67.8 ± 7.7, respectively), and there were no correlations (*p* > 0.05) among echotextural variables determined in original ROIs and protein expression of the inflammatory regulators studied.

**FIGURE 5:**
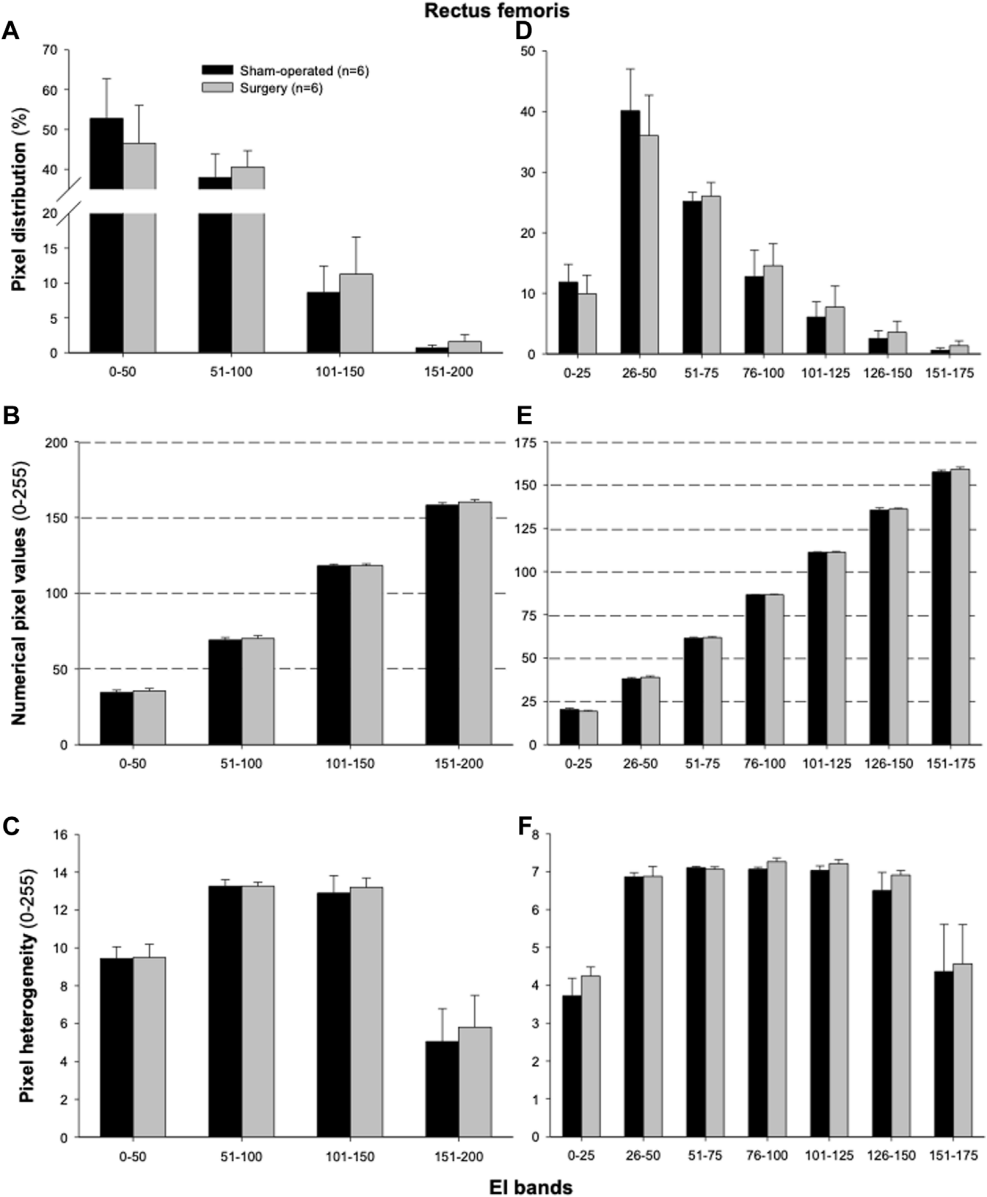
Mean (±SEM) pixel frequency distribution **(A,D)**, intensity [numerical pixel values; **(B,E)**], and heterogeneity **(C,F)** of the rectus femoris muscle ultrasonograms for echointensity (EI) bands with the 50- **(A–C)** or 25-pixel intervals **(D–F)**. Ultrasonographic images of the hindlimbs were taken 3 weeks after the lumbar (L4-L6) facet injury in six sham-operated and six surgically treated rats.

The proportions of pixels (pixel frequency distribution), as well as mean pixel intensity and heterogeneity values for different EI bands, are shown in Figure 5. Only one surgery group rat and no sham-operated animals had pixel intensities for the rectus femoris muscle in the 201–255 range. Similarly, only one sham-operated rat and three surgery rats had pixel intensity values within the 176–200 range. All animals’ ultrasonograms contained numerical pixel values in the lower intensity ranges or bands (≤175). There were no significant differences in the mean pixel intensity and heterogeneity for different EI bands between the surgery and sham-operated groups (Figures 5B, C, E, F). There were no significant correlations (*p* > 0.05) among pixel percentages (Tables 1, 2) or first order echotextural variables in the 50 or 25 EI bands (Tables 3, 4) and the expression of inflammatory regulators studied.

**TABLE 1:** A summary of correlational analyses among pixel frequency distribution (EI%) within individual echointensity bands (EI 0–50 to EI 151–200) and the protein expression of inflammatory mediators measured in the rectus femoris muscle of sham-operated rats and their surgically treated counterparts that underwent experimental lumbar facet (L4-L6) injury.

Expression of inflammatory regulators	EI% (EI band)	*r*	*p*-value
CaMKII	EI% (0–50)	−0.30	0.36
	EI% (51–100)	0.16	0.63
	EI% (101–150)	0.37	0.27
	EI% (151–200)	0.48	0.13
ERK1/2	EI% (0–50)	−0.13	0.71
	EI% (51–100)	0.10	0.78
	EI% (101–150)	0.12	0.72
	EI% (151–200)	0.22	0.51
CGRP	EI% (0–50)	0.04	0.90
	EI% (51–100)	0.19	0.58
	EI% (101–150)	−0.24	0.48
	EI% (151–200)	−0.19	0.57
PAR2	EI% (0–50)	0.06	0.86
	EI% (51–100)	0.08	0.81
	EI% (101–150)	−0.18	0.59
	EI% (151–200)	−0.16	0.64
Substance P	EI% (0–50)	−0.02	0.94
	EI% (51–100)	−0.09	0.79
	EI% (101–150)	0.10	0.78
	EI% (151–200)	0.26	0.43

Abbreviations used are follows: CaMKII, Ca^2^⁺/calmodulin-dependent protein kinase II; ERK1/2, proline-directed kinases; CGRP, calcitonin-gene related peptide; and PAR2, protease-activated receptor-2.

**TABLE 2:** A summary of correlational analyses among pixel frequency distribution (EI%) within individual echointensity bands (EI 0–25 to EI 151–175) and the protein expression of inflammatory mediators measured in the rectus femoris muscle of sham-operated rats and their surgically-treated counterparts that underwent experimental lumbar facet (L4-L6) injury.

Expression of inflammatory regulators	EI% (EI band)	*r*	*p*-value
CaMKII	EI% (0–25)	−0.25	0.46
	EI% (26–50)	−0.30	0.37
	EI% (51–75)	−0.11	0.76
	EI% (76–100)	0.25	0.45
	EI% (101–125)	0.36	0.27
	EI% (126–150)	0.37	0.26
	EI% (151–175)	0.48	0.14
ERK1/2	EI% (0–25)	−0.08	0.81
	EI% (26–50)	−0.15	0.65
	EI% (51–75)	0.03	0.94
	EI% (76–100)	0.11	0.76
	EI% (101–125)	0.12	0.73
	EI% (126–150)	0.12	0.72
	EI% (151–175)	0.22	0.51
CGRP	EI% (0–25)	−0.18	0.60
	EI% (26–50)	0.13	0.69
	EI% (51–75)	0.57	0.07
	EI% (76–100)	−0.05	0.87
	EI% (101–125)	−0.27	0.43
	EI% (126–150)	−0.19	0.58
	EI% (151–175)	−0.17	0.61
PAR2	EI% (0–25)	−0.13	0.69
	EI% (26–50)	0.15	0.66
	EI% (51–75)	0.28	0.41
	EI% (76–100)	−0.04	0.91
	EI% (101–125)	−0.19	0.57
	EI% (126–150)	−0.15	0.65
	EI% (151–175)	−0.15	0.66
Substance P	EI% (0–25)	0.05	0.88
	EI% (26–50)	−0.06	0.86
	EI% (51–75)	−0.14	0.68
	EI% (76–100)	−0.04	0.90
	EI% (101–125)	0.09	0.80
	EI% (126–150)	0.11	0.75
	EI% (151–175)	0.26	0.44

Abbreviations used are follows: CaMKII, Ca^2^⁺/calmodulin-dependent protein kinase II; ERK1/2, proline-directed kinases; CGRP, calcitonin-gene related peptide; and PAR2, protease-activated receptor-2.

**TABLE 3:** A summary of correlational analyses among mean numerical pixel values (NPV) and pixel heterogeneity (SD of numerical pixel values) within individual echointensity bands (EI 0–50 to EI 151–200) and the protein expression of inflammatory mediators measured in the rectus femoris muscle of sham-operated rats and their surgically treated counterparts that underwent experimental lumbar facet (L4-L6) injury.

Expression of inflammatory regulators	NPV (EI band)	*r*	*p*-value	SD (EI band)	*r*	*p*-value
CaMKII	NPV (0–50)	0.31	0.36	SD (0–50)	−0.16	0.64
	NPV (51–100)	0.32	0.34	SD (51–100)	−0.05	0.89
	NPV (101–150)	0.09	0.79	SD (101–150)	0.12	0.73
	NPV (151–200)	0.26	0.44	SD (151–200)	0.23	0.50
ERK1/2	NPV (0–50)	0.10	0.77	SD (0–50)	0.007	0.98
	NPV (51–100)	0.15	0.65	SD (51–100)	0.05	0.87
	NPV (101–150)	−0.06	0.85	SD (101–150)	−0.02	0.96
	NPV (151–200)	0.42	0.20	SD (151–200)	0.11	0.74
CGRP	NPV (0–50)	0.08	0.82	SD (0–50)	−0.02	0.96
	NPV (51–100)	−0.18	0.60	SD (51–100)	0.01	0.97
	NPV (101–150)	0.28	0.40	SD (101–150)	0.31	0.35
	NPV (151–200)	−0.07	0.83	SD (151–200)	−0.06	0.85
PAR2	NPV (0–50)	0.10	0.76	SD (0–50)	−0.12	0.73
	NPV (51–100)	−0.12	0.72	SD (51–100)	−0.11	0.74
	NPV (101–150)	0.18	0.60	SD (101–150)	0.14	0.67
	NPV (151–200)	−0.51	0.11	SD (151–200)	−0.36	0.27
Substance P	NPV (0–50)	−0.005	0.99	SD (0–50)	0.13	0.71
	NPV (51–100)	0.03	0.94	SD (51–100)	−0.10	0.77
	NPV (101–150)	−0.03	0.92	SD (101–150)	0.009	0.98
	NPV (151–200)	0.52	0.10	SD (151–200)	0.19	0.57

Abbreviations used are follows: CaMKII, Ca^2^⁺/calmodulin-dependent protein kinase II; ERK1/2, proline-directed kinases; CGRP, calcitonin-gene related peptide; and PAR2, protease-activated receptor-2.

**TABLE 4:** A summary of correlational analyses among mean numerical pixel values (NPV) and pixel heterogeneity (SD of numerical pixel values) within individual echointensity bands (EI 0–25 to EI 151–175) and the protein expression of inflammatory mediators measured in the rectus femoris muscle of sham-operated rats and their surgically treated counterparts that underwent experimental lumbar facet (L4-L6) injury.

Expression of inflammatory regulators	NPV (EI band)	*r*	*p*-value	SD (EI band)	*r*	*p*-value
CaMKII	NPV (0–25)	−0.31	0.35	SD (0–25)	0.35	0.29
	NPV (26–50)	0.39	0.24	SD (26–50)	−0.21	0.53
	NPV (51–75)	0.35	0.30	SD (51–75)	0.04	0.90
	NPV (76–100)	0.124	0.72	SD (76–100)	0.24	0.47
	NPV (101–125)	0.06	0.85	SD (101–125)	0.12	0.72
	NPV (126–150)	0.158	0.64	SD (126–150)	0.16	0.64
	NPV (151–175)	0.09	0.79	SD (151–175)	0.06	0.87
ERK1/2	NPV (0–25)	−0.28	0.41	SD (0–25)	0.32	0.33
	NPV (26–50)	0.20	0.55	SD (26–50)	−0.07	0.83
	NPV (51–75)	0.16	0.63	SD (51–75)	−0.32	0.34
	NPV (76–100)	0.03	0.92	SD (76–100)	0.54	0.09
	NPV (101–125)	0.04	0.90	SD (101–125)	0.41	0.21
	NPV (126–150)	0.01	0.97	SD (126–150)	0.26	0.45
	NPV (151–175)	0.49	0.12	SD (151–175)	0.11	0.75
CGRP	NPV (0–25)	0.21	0.53	SD (0–25)	−0.31	0.36
	NPV (26–50)	0.003	0.99	SD (26–50)	0.25	0.46
	NPV (51–75)	−0.23	0.50	SD (51–75)	−0.28	0.39
	NPV (76–100)	−0.38	0.24	SD (76–100)	0.02	0.95
	NPV (101–125)	0.09	0.79	SD (101–125)	0.21	0.54
	NPV (126–150)	0.27	0.43	SD (126–150)	0.28	0.41
	NPV (151–175)	0.07	0.83	SD (151–175)	0.08	0.81
PAR2	NPV (0–25)	0.25	0.46	SD (0–25)	−0.30	0.38
	NPV (26–50)	−0.02	0.96	SD (26–50)	0.13	0.71
	NPV (51–75)	−0.16	0.64	SD (51–75)	−0.21	0.53
	NPV (76–100)	−0.51	0.11	SD (76–100)	0.16	0.64
	NPV (101–125)	0.18	0.60	SD (101–125)	0.08	0.81
	NPV (126–150)	0.13	0.70	SD (126–150)	0.09	0.78
	NPV (151–175)	−0.54	0.08	SD (151–175)	−0.43	0.19
Substance P	NPV (0–25)	−0.46	0.16	SD (0–25)	0.45	0.16
	NPV (26–50)	0.13	0.69	SD (26–50)	−0.006	0.99
	NPV (51–75)	0.03	0.94	SD (51–75)	−0.45	0.17
	NPV (76–100)	0.16	0.64	SD (76–100)	0.35	0.29
	NPV (101–125)	0.08	0.82	SD (101–125)	0.41	0.22
	NPV (126–150)	0.06	0.86	SD (126–150)	0.16	0.63
	NPV (151–175)	0.57	0.07	SD (151–175)	0.17	0.62

Abbreviations used are follows: CaMKII, Ca^2^⁺/calmodulin-dependent protein kinase II; ERK1/2, proline-directed kinases; CGRP, calcitonin-gene related peptide; and PAR2, protease-activated receptor-2.

**FIGURE 6:**
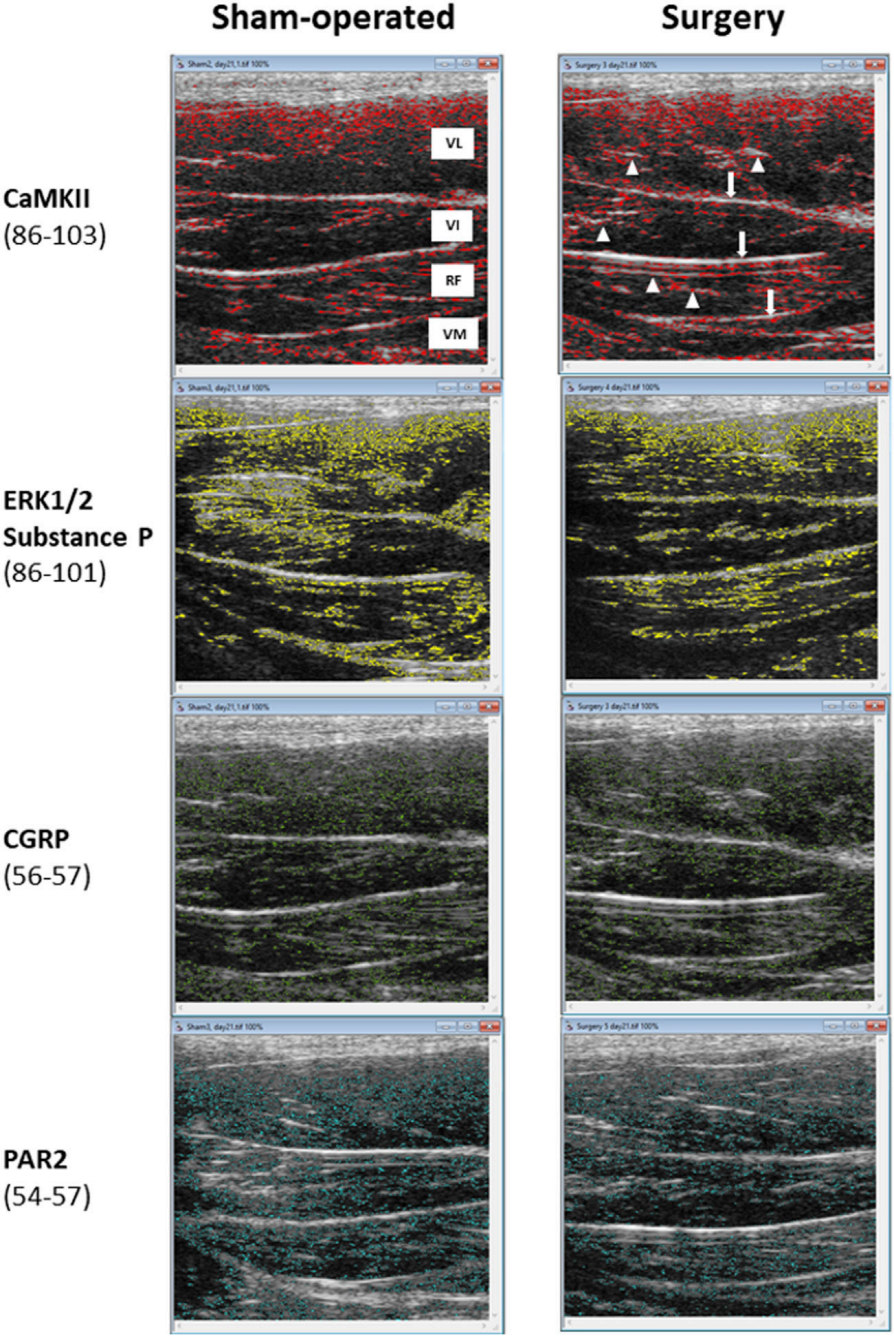
The threshold mapping of specific pixel intensity ranges identified by the r-Algo computer program for which mean numerical pixel values were highly and significantly correlated with the protein expression of neuroinflammatory regulators in the rectus femoris muscle of rats (sham-operated and surgery) after the experimental L4-L6 facet injury. Abbreviations in the top left panel are as follows: VL-vastus lateralis; VI-vastus intermedius; RF-rectus femoris; and VM-vastus medialis. White arrows in a top right panel indicate the perimysial layers and arrowheads point at scattered epimysial septa.

**FIGURE 7:**
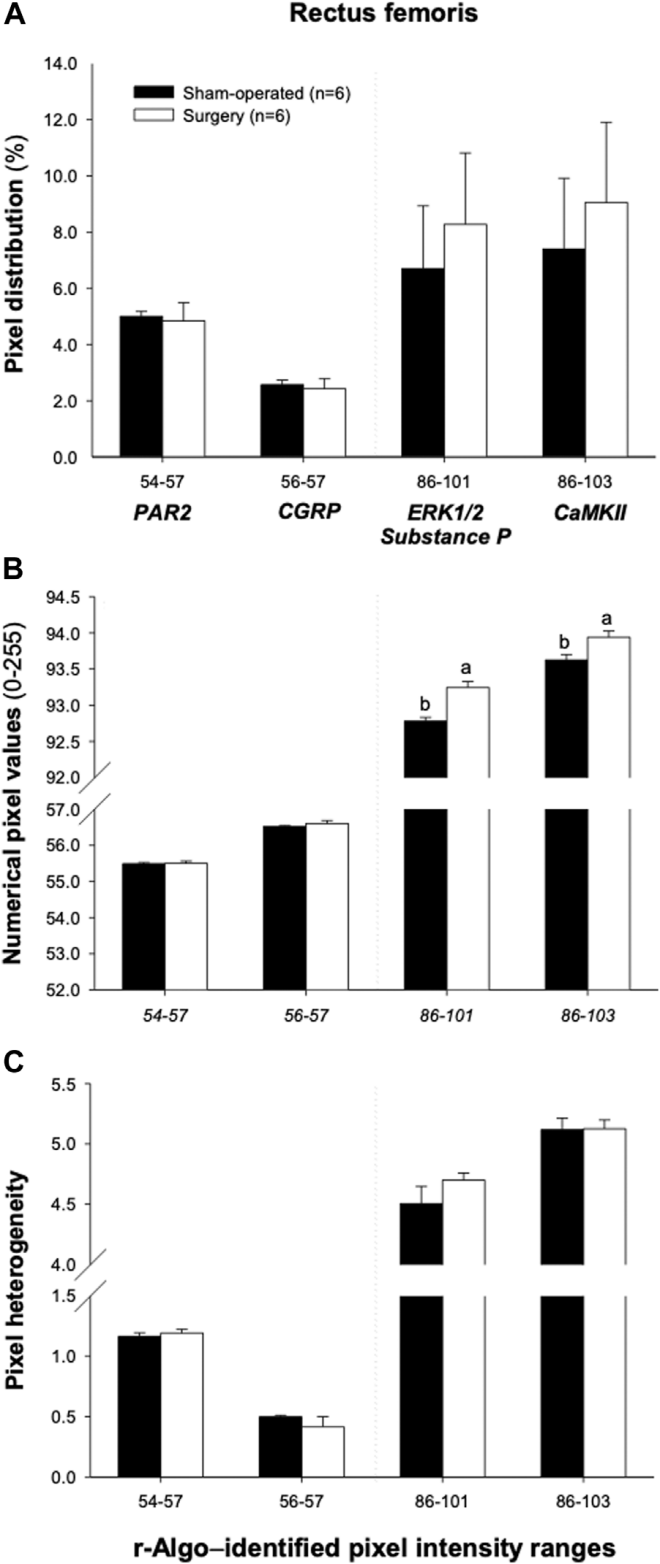
Mean (±SEM) pixel frequency distribution **(A)**, intensity [numerical pixel values; **(B)**], and heterogeneity **(C)** of the rectus femoris muscle ultrasonograms, for specific echointensity (EI) ranges identified by the r-Algo computer program (for which mean numerical pixel values were highly and significantly correlated with the protein expression of neuroinflammatory regulators). Ultrasonographic images of the hindlimbs were obtained in 6 sham-operated and 6 surgery rats that underwent the experimental L4-L6 facet injury. Abbreviations used are follows: Ca^2^⁺/calmodulin-dependent protein kinase II (CaMKII); proline-directed kinases (ERK1/2); calcitonin-gene related peptide (CGRP); and protease-activated receptor-2 (PAR2).

The ranges of pixel intensities identified with the r-Algo program, for which there were the strongest linear relationships with the protein expression of inflammatory mediators measured in the rectus femoris of surgery and sham-operated rats, were as follows: PAR2: 54–57; CGRP: 56–57; ERK1/2 and substance P: 86–101, and CaMKII: 86–103. Images illustrating the spatial distribution of pixels within the pixel ranges above are given in Figure 6. There were no differences (*p* > 0.05) in the percentages of pixels within specific ranges (relative to all pixels in ROI) between the surgery and sham-operated groups (Figure 7A). Mean NPVs for the pixel ranges corresponding to ERK1/2 and substance P (86–101) and CaMKII (86–103) were greater (*p* < 0.05) in surgery rats compared with their sham-operated counterparts (Figure 7B). However, no significant differences were observed for pixel heterogeneity values of any specific pixel range (Figure 7C). There were no significant correlations among the percentages of pixels in the pixel ranges determined with the r-Algo and the expression levels of inflammatory mediators (Table 5). Pearson Product Moment analyses revealed a lack of linear relationships among NPV values in the non-corresponding pixel ranges and the expression of inflammatory regulators. There were no significant correlations among pixel heterogeneity and the expression of inflammatory regulators except for pixel heterogeneity SD (56–57) and CGRP protein expression (*r* = −0.94, *p* = 0.00002) and SD (56–57; an NPV region partially overlapping the 54–57 region specific for PAR2) and PAR2 protein expression in the rectus femoris of rats (*r* = −0.78, *p* = 0.004; Table 6).

**TABLE 5:** A summary of correlational analyses among pixel frequency distribution (EI%) within individual echointensity bands identified by the r-Algo program and the protein expression of inflammatory mediators measured in the rectus femoris muscle of sham-operated rats and their surgically treated counterparts that underwent experimental lumbar facet (L4-L6) injury.

Expression of inflammatory regulators	r-Algo EI band	*r*	*p*-value
CaMKII	EI% (54–57)	−0.45	0.16
	EI% (56–57)	−0.25	0.46
	EI% (86–101)	0.33	0.32
	*EI% (86*–*103)*	0.33	*0.32*
ERK1/2	EI% (54–57)	0.05	0.88
	EI% (56–57)	−0.05	0.88
	*EI% (86*–*101)*	0.16	*0.64*
	EI% (86–103)	0.15	0.67
CGRP	EI% (54–57)	0.41	0.21
	*EI% (56*–*57)*	−0.21	*0.53*
	EI% (86–101)	−0.14	0.67
	EI% (86–103)	−0.16	0.64
PAR2	*EI% (54*–*57)*	0.06	*0.86*
	EI% (56–57)	−0.52	0.10
	EI% (86–101)	−0.12	0.72
	EI% (86–103)	−0.12	0.72
Substance P	EI% (54–57)	0.12	0.72
	EI% (56–57)	0.08	0.82
	*EI% (86*–*101)*	0.06	*0.86*
	EI% (86–103)	0.04	0.89

EI band ranges specific for the individual inflammatory mediators listed in the first column are indicated with italics. Abbreviations used are follows: CaMKII: Ca^2^⁺/calmodulin-dependent protein kinase II; ERK1/2: proline-directed kinases; CGRP: calcitonin-gene related peptide; and PAR2: protease-activated receptor-2.

**TABLE 6:** A summary of correlational analyses among mean numerical pixel values (NPV) and pixel heterogeneity (SD of numerical pixel values) within individual echointensity bands identified by the r-Algo program and the protein expression of inflammatory mediators measured in the rectus femoris muscle of sham-operated rats and their surgically treated counterparts that underwent experimental lumbar facet (L4-L6) injury.

Expression of inflammatory regulators	NPV (r-Algo EI band)	*r*	*p*-value	SD (r-Algo EI band)	*r*	*p*-value
CaMKII	NPV (54–57)	0.24	0.48	SD (54–57)	0.18	0.60
	NPV (56–57)	−0.02	0.96	SD (56–57)	−0.04	0.92
	NPV (86–101)	0.65	0.03	SD (86–101)	0.39	0.23
	*NPV (86*–*103)*	0.77	0.005	SD (86–103)	0.27	0.42
ERK1/2	NPV (54–57)	0.02	0.94	SD (54–57)	0.06	0.87
	NPV (56–57)	0.25	0.46	SD (56–57)	−0.25	0.46
	*NPV (86-101)*	0.87	0.0005	SD (86–101)	0.44	0.18
	NPV (86–103)	0.53	0.09	SD (86–103)	−0.07	0.83
CGRP	NPV (54–57)	−0.70	0.02	SD (54–57)	0.12	0.72
	*NPV (56*–*57)*	0.94	*0.00001*	SD (56–57)	−0.94	0.00002
	NPV (86–101)	0.02	0.95	SD (86–101)	−0.15	0.66
	NPV (86–103)	−0.26	0.45	SD (86–103)	−0.34	0.31
PAR2	*NPV (54*–*57)*	−0.82	*0.002*	SD (54–57)	−0.06	0.86
	NPV (56–57)	0.69	0.02	SD (56–57)	−0.78	0.004
	NPV (86–101)	−0.03	0.94	SD (86–101)	−0.33	0.32
	NPV (86–103)	−0.16	0.63	SD (86–103)	−0.45	0.17
Substance P	NPV (54–57)	0.15	0.65	SD (54–57)	−0.09	0.79
	NPV (56–57)	0.11	0.754	SD (56–57)	−0.07	0.83
	*NPV (86*–*101)*	0.78	*0.005*	SD (86–101)	0.49	0.12
	NPV (86–103)	0.46	0.15	SD (86–103)	0.05	0.89

EI band ranges specific for the individual inflammatory mediators listed in the first column are indicated with italics. Abbreviations used are follows: CaMKII, Ca^2^⁺/calmodulin-dependent protein kinase II; ERK1/2, proline-directed kinases; CGRP, calcitonin-gene related peptide; and PAR2, protease-activated receptor-2.

## Discussion

Transcutaneous ultrasonography has evolved significantly over the past decade. The advent of high-frequency micro-ultrasound systems enabled detection of a wide range of pathological muscle conditions, not only in clinical settings but also in non-clinical fields, particularly in studies involving small laboratory rodents [11, 19, 20]. Although ultrasound technique offers a secure means to observe the development or progression of the disease and to assess the effectiveness of treatment(s) applied, only a limited number of studies have explored the functional and morphological aspects of rodent skeletal muscles using ultrasonography [19, 20]. Despite their highly organized microarchitecture, striated muscles exhibit significant structural heterogeneity caused mainly by varying proportions of different muscle fiber types [21], fascial tissue [22, 23], innervation [24], and blood/lymphatic drainage [25]. Changes in muscle echogenicity could be related to microscopic changes occurring in any of these compartments [26]. A major difficulty in detecting and/or quantifying factors critical to inflammatory outcomes in skeletal muscles lies in the complexity of their pathomorphological arrangement. Methodical analysis of ultrasongrams is crucial for achieving proper standardization in non-medical and medical ultrasonography (i.e., enhancing the interpretation of preclinical data and thus facilitating the broader application of ultrasound imaging in clinical settings).

Muscle echogenicity is frequently used to evaluate its structural and functional integrity [19]. Several methods can be employed to assess muscle echotextural parameters. The simplest method is to visually compare the echogenicity of the muscle to that of other structures, such as subcutaneous tissue [19]. The results obtained using this approach are often imprecise due to the potential human error interpreting the differences among multiple “shades of grey” [27]. Semi-quantitative method described by Heckmatt [6] and comparing muscle echointensity with bone echogenicity has greater sensitivity to accurately diagnose pathological conditions of skeletal muscles compared with visual assessment. However, quantitative methods utilizing the computation of echointensity values in a region of interest (ROI) have proven to be the most sensitive (90 percent overall) in detecting an array of neuromuscular disorders [6], including neuromuscular diseases (NMDs) in children [11, 28], Duchenne and Becker muscular dystrophy [29], Floppy disease [30], and spina bifida aperta [31].

Recently, Pinto and Pinto [13] have shown that assessing the concentration of pixels within different clusters of pixel echointensities (EI bands) of the grey-scale histogram can provide more accurate information on muscle constituents than the whole-image quantitative analysis. In contrast to the results of Pinto and Pinto [13], we found no significant differences in pixel echointensity, heterogeneity and distribution concentration in the rectus femoris muscle (neurosegmentally-linked myotomes) between the sham and surgery groups of rats when using the EI bands technique (with either 50- or 25-pixel intervals). Clearly, partitioning the “pixel pallete” into five or eleven bands did not “capture” or “sequester” pixel intensity values associated with the changes in an affected myotome.

Alternatively, a new method of image processing and analysis using the r-Algo, an algorithm written in Python, did identify specific ranges of pixel intensities for which mean numerical pixel values of rectus femoris were strongly correlated (r ≥ 0.77) [32] with the protein expression of inflammatory mediators determined in our study. Mean pixel intensities for the identified regions that corresponded to inflammatory regulators differing significantly between sham-operated and surgery rats, also varied between the two subsets of animals. However, pixel frequency distribution and heterogeneity for these ranges did not vary between the surgery and sham-operated groups, and with the two exceptions (SD (54–57) vs. CGRP and SD (56–57) vs. PAR2) they did not relate to the expression of inflammatory regulators studied. Moreover, the specific EI ranges were identical for substance P and ERK1/2 (86–101), and there was a considerable overlap between the ERK1/2-substance P range (86–101) and that for CaMKII (86–103). This is intriguing since not only were the three inflammatory factors the only ones whose expression was affected 3 weeks after the experimental facet injury, but they also function synergistically during the course of neurogenic inflammation. Substance P has been shown to regulate the activation of inflammatory signaling pathways via ERK1/2 activation in macrophages, neutrophils, smooth muscle cells and muscle lymphatics, and this cascade of intracellular events mediated jointly by substance and ERK1/2 is dependent on intracellular increase in calcium [33]. Changes in p-CaMKII expression result in altered activity of the sarcoendoplasmic reticulum calcium ATPase (SERCA) pump involved in Ca^2+^ transport in muscle cells [34]. Both CGRP and PAR2, albeit exhibiting similar expression levels in the rectus femoris of surgery and sham-operated rats, also had overlapping r-Algo identified pixel intensity ranges, and their role during the neurogenic inflammation is clearly synergistis: activation of PAR2 stimulates the release of SP and CGRP from sensory nerve endings, reinforcing the neurogenic inflammation milieu [35, 36]. Collectively, these observations can be interpreted to suggest that range-specific pixel luminosity values were primary echotextural variables altered in response to the specific alterations elicited in the skeletal muscles of experimental rats by individual inflammatory regulators.

Additional ultrasonogram mapping was performed in an attempt to determine the spatial distribution of the pixels in specific ranges within the ultrasonographic images of the rectus femoris muscle. By performing the threshold mapping using ImageProPlus^®^ program, the pixels specific for CaMKII, ERK1/2 and substance P were observed mainly near the fibroadipose septa of the muscle. It has been shown that inflammatory processes not only alter the echointensity of muscle fascicles, but also the echointensity of fibroadipose septa as they fill with inflammatory exudates [37]. Furthermore, perimysium and epimysium (fibroadipose septa) contain nerve endings that release neuropeptides, including substance P, and it has been shown that substance P stimulates transforming growth factor-1 (TGF-1), which leads to the development of fibrotic tissue in those regions of the muscle [38]. Even though it is only a speculation, it is attractive to suggest that r-Algo-aided detection of numerical pixel values associated with specific biochemical constituents may serve as an indicator of their locality and/or the extent of histomorphological changes induced by their elevated expression or accumulation.

## Conclusion

Our present results indicate that identifying specific pixel intensity ranges was necessary to find significant quantitative correlations between mean numerical pixel values and protein expression of inflammatory mediators (related to putative microstructural changes in the neurosegmentally-linked skeletal muscles of rats after experimental facet injury). The application of this method of image processing and analysis may enable the extraction of critical information on quantitative relationships among first-order ultrasonographic image attributes and an array of output variables (e.g., chemical constituents or histomorphological features of the ultrasonographically examined tissue of interest). This potential feature of r-Algo warrants further studies.

## Data Availability

The original contributions of the study are included in the article/supplementary material. Further inquiries can be directed to the corresponding author.
